# Employment outlook before search? A four-wave RI-CLPM study of environmental career exploration and perceived employment outlook among undergraduates in Liaoning Province, China

**DOI:** 10.3389/fpsyg.2026.1921600

**Published:** 2026-09-04

**Authors:** Yehong Fan, Zhen Huang

**Affiliations:** 1 School of Foreign Languages, Liaoning Normal University, Dalian, Liaoning, China; 2 School of Physical Education, Liaoning Normal University, Dalian, Liaoning, China

**Keywords:** career self-management, Liaoning Province, environmental career exploration, perceived employment outlook, random-intercept cross-lagged panel model, within-person dynamics

## Abstract

Career exploration is often expected to improve university students’ employment expectations, whereas favorable expectations may also encourage subsequent career action. This four-wave longitudinal study examined between-person and within-person associations between environmental career exploration (ECE) and perceived employment outlook (EPO) among 2,267 undergraduates recruited from 16 universities in Liaoning Province, China. Surveys were administered across one academic year at intervals of approximately 8, 13, and 9 weeks. A free-lag random-intercept cross-lagged panel model (RI-CLPM) with demographic and institutional variables included as missing-data auxiliaries separated stable between-person differences from occasion-specific deviations. The random intercepts were positively correlated, r = 0.673, *p* < 0.001. Higher-than-usual EPO predicted higher subsequent ECE from T1 to T2 (*β* = 0.065, *p* = 0.030) and from T3 to T4 (*β* = 0.094, *p* = 0.001), but not across the longer winter-recess interval from T2 to T3 (*β* = 0.022, *p* = 0.482). Higher-than-usual ECE predicted lower subsequent EPO only from T3 to T4 (*β* = −0.069, *p* = 0.024); the earlier paths were nonsignificant. Multiple-indicator modeling that accommodated the noninvariant EPO3 intercepts and a two-item EPO sensitivity analysis produced the same interval-specific pattern. Stratified comparisons showed no university-tier or academic-major differences that survived multiplicity correction. The findings indicate that the prospective ECE–EPO association varied across the academic year in this regional sample. Because ECE measured search frequency rather than the content or valence of information obtained, the negative final-interval association does not establish that exploration is ineffective or identify the mechanism underlying expectation revision.

## 1 Introduction

The transition from university to employment requires students to act before employment outcomes are known. They must learn about occupations, industries, organizations, and labor-market conditions while simultaneously forming judgments about whether desirable employment is attainable. Two constructs are therefore particularly important during this preparatory period. Environmental career exploration (ECE) refers to purposeful efforts to obtain information about occupations, jobs, organizations, and the external career environment (Stumpf et al., 1983). Perceived employment outlook (EPO), by contrast, refers to students’ subjective assessment of their prospects of obtaining suitable, preferred, and interesting work under prevailing labor-market conditions (Hodzic et al., 2015). ECE represents career action, whereas EPO represents a context-sensitive career-related expectation about the attainability of future employment (Akkermans et al., 2024; Antonio and Chiesa, 2024).

These constructs are likely to be connected, but their temporal ordering remains theoretically unresolved. A common assumption in higher education and career-development practice is that students become more confident about their employment prospects by exploring careers. Yet the opposite process is also plausible: students who believe that desirable employment is attainable may be more willing to invest effort in career exploration. Moreover, exploration does not necessarily improve employment confidence. Information acquired through exploration may reveal previously unknown opportunities, but it may also expose competition, qualification requirements, occupational constraints, and discrepancies between personal preferences and available jobs. Career exploration may therefore strengthen, weaken, or leave unchanged students’ subsequent employment outlook (Grosemans et al., 2024; Haenggli et al., 2025; Kleine et al., 2021; Ma et al., 2024).

Clarifying these possibilities requires a dynamic, within-person perspective. The relevant question is whether a student who becomes more optimistic than usual subsequently explores more than usual, and whether a temporary increase in that student’s exploration is followed by a change in the student’s own employment outlook. Recent RI-CLPM research on self-perceived employability further illustrates why stable between-person differences and within-person fluctuations should be distinguished (Petruzziello et al., 2025). The present four-wave study examines these between-person and within-person relations among undergraduates enrolled at participating universities in Liaoning Province (Hamaker et al., 2015; Orth et al., 2024; Rohrer and Murayama, 2023; Usami, 2021).

The regional setting defines the scope of the study. Recent employment initiatives in Liaoning have emphasized employer–university matching, adjustment of programs with persistently weak employment outcomes, and short training or micro-program options for students in fields with insufficient social demand (Liaoning Provincial Department of Education, 2025; Liaoning Provincial Department of Human Resources and Social Security, 2025). These priorities indicate a regionally differentiated employment environment rather than a homogeneous national labor market. The study did not assess students’ intended work locations or their perceptions of provincial employment policy; the regional setting is therefore treated as a boundary condition, not as a tested mechanism.

### 1.1 Environmental career exploration and perceived employment outlook

Career exploration is a central career self-management process through which individuals acquire and process information relevant to their future work (Flum and Blustein, 2000; Jiang et al., 2019). It can involve reflection on personal interests, values, and capabilities, as well as investigation of the external career environment. The present study focuses specifically on environmental exploration because it most directly captures students’ encounters with labor-market information. ECE includes behaviors such as investigating occupations, learning about organizations, gathering information about job requirements, consulting people with occupational experience, and examining potential employment opportunities (Stumpf et al., 1983). Unlike relatively stable personal characteristics, these behaviors can increase or decrease across relatively short periods as students encounter new information, demands, and opportunities (Morgan et al., 2024).

Research generally portrays career exploration as an adaptive developmental behavior. It can help students clarify alternatives, build labor-market knowledge, and prepare for decisions, although its consequences vary by exploration dimension and context (Kleine et al., 2021). Recent longitudinal work shows that environmental exploration can precede changes in career knowledge and decidedness, while self-regulated learning can help translate proactive career behavior into more sustainable career outcomes (Haenggli et al., 2025; Morgan et al., 2024; Qalati et al., 2025). These findings support a process view of exploration without implying that more frequent search automatically improves every employment-related appraisal.

EPO concerns a different component of career development. Perceived employability has broadly been defined as an individual’s perception of the possibility of obtaining and maintaining employment (Vanhercke et al., 2014). Contemporary research treats employability as a contextual and developmental appraisal shaped by personal resources, educational experiences, institutional conditions, and labor-market opportunities or barriers (Akkermans et al., 2024; Ananthram et al., 2024; Antonio and Chiesa, 2024). Multidimensional student measures can therefore combine opportunity expectations with comparatively stable resource and readiness components. Such measures are useful for describing differences between students, but they may be less sensitive to short-term changes within the same student. The present study therefore adopts a narrower opportunity-oriented construct. EPO captures students’ current appraisal of whether they are likely to obtain employment that is suitable, personally desirable, and interesting given current labor-market conditions (Hodzic et al., 2015).

Recent studies continue to link exploration, future orientation, mentoring, or career engagement with perceived employability and related future-oriented appraisals, including evidence among Chinese undergraduates and longitudinal evidence among recent graduates (Chen et al., 2023; Grosemans et al., 2024; Ma et al., 2024; Niu et al., 2025). Students who characteristically explore more may acquire more information and resources, but they may also differ in motivation, self-regulation, work experience, social support, institutional resources, and career preparedness (Antonio and Chiesa, 2024; Brosnan et al., 2024; Paganin et al., 2025). Accordingly, a positive between-person association does not show that increasing exploration within a particular student will subsequently improve that student’s employment outlook (Akkermans et al., 2024; Gerçek, 2024; Petruzziello et al., 2025).

Nevertheless, the accumulated evidence suggests that students who are generally more optimistic about their employment opportunities are also likely to be more engaged in exploring their career environment. Accordingly, the following between-person hypothesis was proposed:

*H1*: At the between-person level, the stable between-person component of ECE will be positively associated with the stable between-person component of EPO.

### 1.2 Perceived employment outlook as an antecedent of environmental career exploration

The social cognitive model of career self-management provides a theoretical basis for viewing employment-related beliefs as antecedents of career behavior. This model proposes that adaptive career behaviors are shaped by self-efficacy beliefs, outcome expectations, goals, and contextual supports or barriers (Lent and Brown, 2013). Individuals are more likely to pursue career-management activities when they believe that desirable career outcomes are attainable and that their effort can contribute to those outcomes. Empirical applications of the model have shown that positive social-cognitive beliefs support intentions and engagement in career exploration and decision-making behaviors (Lent et al., 2016, 2019; Eccles and Wigfield, 2020).

EPO is not identical to exploration-specific self-efficacy or behavior-specific outcome expectations. It does not assess whether students believe that they can perform a particular search task or the expected benefit of one specific action. Its motivational direction is nevertheless comparatively clear: a temporary improvement in EPO can increase the perceived attainability of a valued distal outcome and the anticipated usefulness of searching for information. Search effort is more likely to appear worthwhile when students can connect it to plausible employment pathways. Recent research similarly links future-oriented appraisals and career decision confidence with adaptive career processes and shows that proactive behavior acquires developmental value through self-regulatory mechanisms (Badwy et al., 2025; Qalati et al., 2025). EPO may therefore support approach-oriented engagement while remaining conceptually distinct from self-efficacy.

A less favorable outlook may have a different motivational effect. Students who temporarily perceive few viable employment opportunities may conclude that further exploration is unlikely to be beneficial, experience greater uncertainty or discouragement, or postpone engagement with career-related information. Although a difficult employment environment could motivate compensatory action in some students, low expectations can also reduce goal investment when the expected return from effort appears limited. Recent evidence also shows that economic constraints and career barriers can undermine perceived employability, while contextual resources may buffer those associations (Kirazci, 2026; Rodrigues et al., 2024). From a within-person perspective, a student’s temporary movement above or below their usual EPO may therefore precede corresponding changes in career exploration (Eccles and Wigfield, 2020).

Recent longitudinal findings support the possibility that employment-related appraisals precede career behavior. Grosemans et al. (2024) found that changes in perceived employability preceded changes in broad career engagement, whereas the reverse path was not supported. Recent research also links future employability, career adaptability, self-regulation, and career motivation (Ayvaz and Elhatip, 2026; Azalea et al., 2026; Badwy et al., 2025; Qalati et al., 2025). Because these constructs are broader than ECE, the within-person relation between EPO and environmental information seeking remains open.

The present study tests this process at the within-person level. A positive cross-lagged association would mean that when the same student reports a more favorable employment outlook than usual at one wave, that student subsequently engages in more environmental career exploration than usual. Therefore:

*H2*: At the within-person level, higher-than-usual EPO at a given wave will predict higher-than-usual ECE at the subsequent wave.

### 1.3 Environmental career exploration as a precursor of employment outlook: opportunity discovery or expectation revision?

The reverse pathway from ECE to EPO is theoretically less straightforward. One possibility is an opportunity-discovery process. Exploration provides access to information that was previously unavailable or insufficiently processed (Stumpf et al., 1983). By learning about a wider range of occupations, employers, employment routes, and qualification requirements, students may identify opportunities that they had not previously considered. They may also develop clearer links between their academic experience and possible jobs, discover multiple routes toward a desired occupational outcome, and gain a more differentiated understanding of the labor market. In this way, exploration can reduce ambiguity and increase the perceived attainability of suitable work (Flum and Blustein, 2000; Jiang et al., 2019).

This positive account is consistent with developmental views of exploration as an adaptive process. Career exploration can support learning, career clarity, and preparation for decision-making (Flum and Blustein, 2000; Jiang et al., 2019). Recent longitudinal evidence indicates that increases in environmental exploration can precede increases in labor-market knowledge and career decidedness, suggesting that exploratory action can generate career-relevant cognitive resources (Haenggli et al., 2025). Recent cross-sectional, time-lagged, and longitudinal studies also report links among career exploration or engagement, self-regulated learning, and employability-related appraisals (Grosemans et al., 2024; Ma et al., 2024; Morgan et al., 2024; Tuononen et al., 2024). From this perspective, a student who explores more than usual might subsequently perceive more, rather than fewer, viable employment possibilities.

However, exploration is fundamentally an information-acquisition process, not an intervention designed solely to increase confidence. New information can revise expectations upward or downward. Students may begin with incomplete or overly favorable assumptions about job availability, qualification requirements, salaries, geographic mobility, or the competitiveness of preferred occupations. More intensive exploration may reveal that desired positions are scarce, that employers require credentials or experience the student has not yet acquired, or that available work is inconsistent with the student’s preferences. Recent studies likewise show that perceived employability and career expectations are shaped by economic constraints, career barriers, and configurations of personal and contextual resources (Antonio and Chiesa, 2024; Kirazci, 2026; Paganin et al., 2025; Rodrigues et al., 2024). In these circumstances, exploration may produce a less favorable but more information-based employment outlook.

The directional uncertainty of the ECE-to-EPO pathway arises because ECE records how frequently students search, not what they learn. A positive coefficient would be consistent with opportunity discovery; a negative coefficient would be consistent with downward expectation revision. A nonsignificant coefficient, however, would not identify a balance between the two processes. Testing that account would require direct measures of information valence, credibility, relevance, and interpretation.

Existing evidence does not resolve these competing possibilities. Positive associations in cross-sectional studies may indicate that exploration improves employment perceptions, but they may also arise because optimistic and career-ready students engage in more exploration. The time-lagged study by Ma et al. (2024) provides stronger temporal evidence, but a two-wave design cannot fully distinguish stable between-person differences from within-person change. In contrast, the study by Grosemans et al. (2024) found no evidence that changes in broad career engagement produced subsequent changes in perceived employability during the graduate transition. Yet broad career engagement is not equivalent to ECE, and recent graduates differ developmentally from undergraduates who are still constructing initial labor-market expectations (Cole and Maxwell, 2003; Dormann and Griffin, 2015; Hamaker et al., 2015; Usami, 2021).

Because theory supports both opportunity discovery and downward expectation revision, imposing a single directional prediction would be premature. The present study therefore addresses the following research question:

Research Question 1. At the within-person level, does higher-than-usual ECE at a given wave predict subsequent EPO, and does the association vary across adjacent intervals?

A negative association would indicate downward revision in EPO, not more accurate calibration, because accuracy requires comparison with objective employment outcomes. A nonsignificant average coefficient would indicate that no reliable average prospective association was detected at the observed interval; it would not establish that exploration is irrelevant or reveal whether unmeasured positive and negative informational experiences differed across students or occasions.

### 1.4 The present study

The present research extends the career exploration and perceived-employability literature in four focused ways. It examines ECE as the frequency of engagement with the external career environment and EPO as a subjective appraisal of attainable post-graduation work. Keeping the constructs narrow avoids combining exploration with other career-management behaviors or combining current employment expectations with institutional reputation and accumulated human capital.

It also develops an asymmetric theoretical rationale. EPO has a comparatively direct motivational implication because perceived attainability can change the anticipated value of career action. ECE has a less determinate implication for EPO because search frequency does not specify whether the information encountered is encouraging, discouraging, credible, or useful. The study therefore tests a positive EPO-to-ECE hypothesis and treats the reverse pathway as a nondirectional, interval-specific research question.

The RI-CLPM separates stable between-person differences from occasion-specific within-person deviations (Hamaker et al., 2015). This distinction permits a more accurate test of temporal ordering than a conventional CLPM, although it does not identify causal effects or eliminate time-varying confounding (Muthén and Asparouhov, 2024; Rohrer and Murayama, 2023).

The study focuses on undergraduates enrolled at participating universities in Liaoning Province before the immediate graduation transition. The three adjacent intervals were approximately 8, 13, and 9 weeks, and the middle interval crossed the winter recess. Lagged coefficients were therefore estimated freely in the primary model. Stratified analyses also examined whether the prospective associations differed by university tier and academic major.

Research Question 2. Do the within-person cross-lagged associations between ECE and EPO differ between students at Double First-Class and non-Double First-Class universities?

Research Question 3. Do the within-person cross-lagged associations between ECE and EPO differ between STEM and humanities and social sciences students?

**Figure 1:**
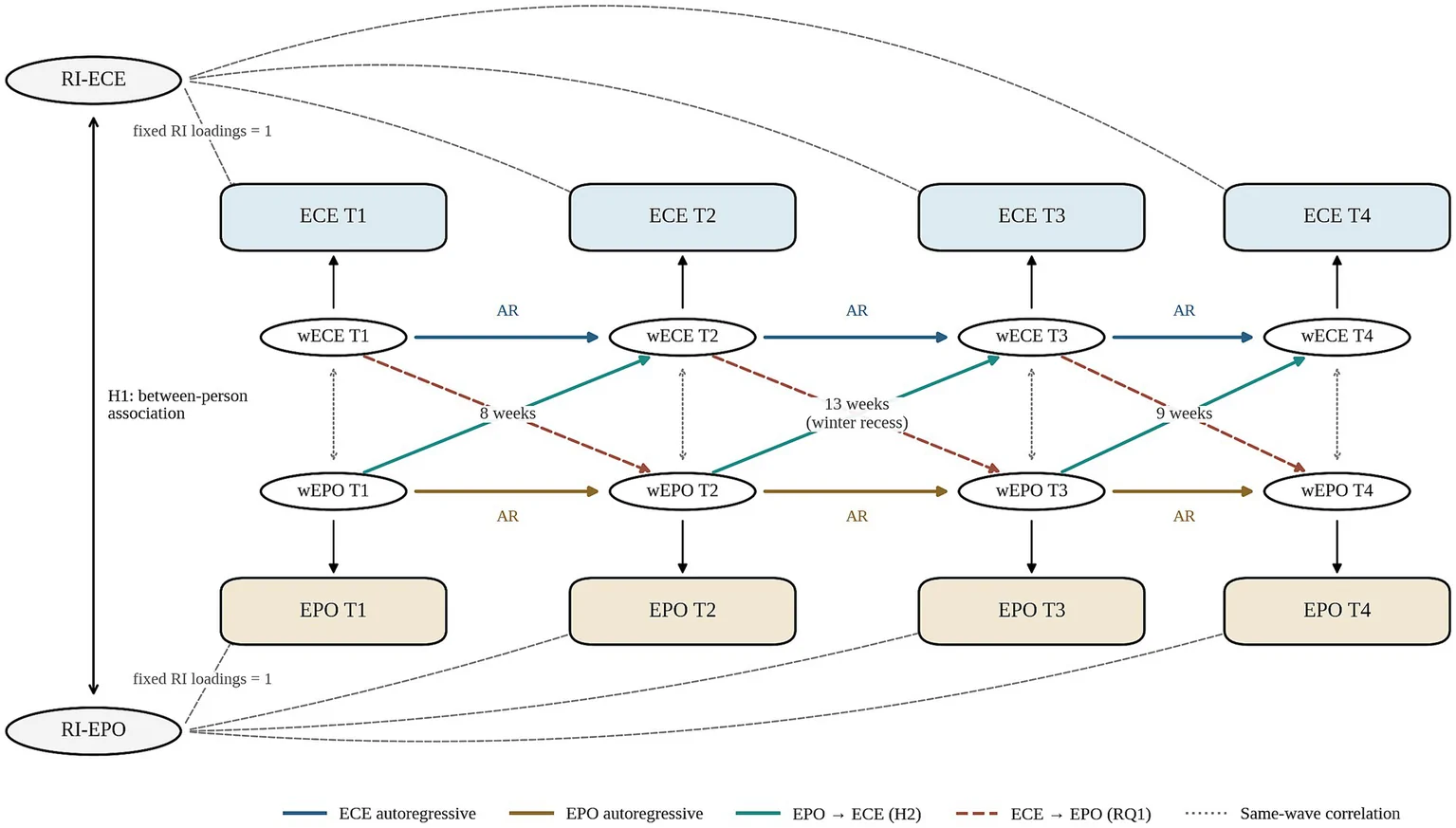
Theoretical free-lag random-intercept cross-lagged panel model of environmental career exploration and perceived employment outlook. ECE, environmental career exploration; EPO, perceived employment outlook; RI-ECE and RI-EPO, random intercepts representing stable between-person differences; wECE and wEPO, occasion-specific within-person deviations; AR, autoregressive path. Random-intercept and within-person factor loadings are fixed at 1. The EPO-to-ECE pathway is hypothesized to be positive; the ECE-to-EPO pathway is examined as a nondirectional research question. The approximate intervals were 8, 13, and 9 weeks, with the middle interval spanning the winter recess.

Figure 1 presents the theoretical model. Hypothesis 1 concerns the correlation between the stable random intercepts. Hypothesis 2 concerns positive EPO-to-ECE paths at the within-person level. The reciprocal ECE-to-EPO paths address Research Question 1, and the stratified comparisons address Research Questions 2 and 3.

## 2 Materials and methods

### 2.1 Participants and procedure

This study used a four-wave longitudinal panel design to examine reciprocal prospective associations between ECE and EPO among undergraduates enrolled at 16 universities in Liaoning Province, China. The participating institutions included Double First-Class universities, other public undergraduate universities, and private undergraduate universities and represented several disciplinary profiles. Data were collected from October 2025 to May 2026: T1, October 13–24; T2, December 8–19; T3, March 9–20; and T4, May 11–22. Based on the midpoints of the data-collection windows, adjacent intervals were approximately 8, 13, and 9 weeks. The T2–T3 interval spanned the winter recess. Because the calendar intervals differed and the middle interval represented a distinct academic context, the primary structural model estimated adjacent lagged paths freely rather than assuming equal effects across the academic year.

At baseline, 2,531 paper-and-pencil questionnaires were distributed, and 2,411 were returned, yielding a response rate of 95.26%. After data-quality screening, 2,267 valid baseline questionnaires were retained, corresponding to 89.57% of all distributed questionnaires and 94.03% of all returned questionnaires. The baseline analytic sample therefore consisted of 2,267 undergraduate students aged 18 years or older. All participants were adults, and the first page of the questionnaire explicitly stated that only students aged 18 years or older were eligible to participate. The page also described the study purpose, voluntary nature of participation, confidentiality protections, approximate completion time, and the right to withdraw at any time without penalty. Written informed consent was obtained from all participants before questionnaire administration. To link responses across waves while protecting anonymity, participants generated a confidential self-identification code based on the instructions printed on the questionnaire; names, student identification numbers, and phone numbers were not retained in the research dataset. The study protocol was reviewed and approved by the Ethics Committee of the Liaoning Normal University.

Questionnaires were administered offline in classroom or campus settings by trained research assistants. Before each administration, students were informed that there were no right or wrong answers, that individual responses would be used only for academic research, and that teachers or university administrators would not have access to individual-level data. The research assistants were present to answer procedural questions but did not explain or interpret substantive items. To improve response quality, the questionnaire emphasized anonymity, voluntary participation, and careful reading. One instructed-response attention-check item was embedded in the questionnaire: “To show that you are reading carefully, please select ‘Agree’ for this item.” Instructed-response items are widely used to identify insufficient-effort or careless responding in survey research (Huang et al., 2012; Meade and Craig, 2012).

Invalid baseline questionnaires were excluded according to prespecified quality-control criteria. Specifically, questionnaires were treated as invalid if the participant did not provide written informed consent, reported being younger than 18 years old, failed the attention-check item, had excessive missing responses on the focal measures, showed an implausible invariant response pattern across substantive items, or provided information that made longitudinal matching impossible. In total, 144 returned baseline questionnaires were excluded during screening, resulting in the valid baseline sample of 2,267 participants. The demographic characteristics of the baseline analytic sample are presented in Table 1 (Huang et al., 2012; Meade and Craig, 2012).

**Table 1:** Baseline demographic characteristics of the analytic sample (*N* = 2,267).

Characteristic	Category	*n*	Percent
Gender	Male	1,054	46.49
	Female	1,208	53.29
	Other/Prefer not to say	5	0.22
Major	Science, Technology, Engineering and Mathematics	964	42.52
	Humanities and Social Sciences	558	24.61
	Business, Economics and Management	530	23.38
	Arts and Design	215	9.48
University tier	Double First-Class University	342	15.09
	Non-Double First-Class University	1,925	84.91
Institution type	Double First-Class university	342	15.09
	Other public undergraduate university	1,563	68.95
	Private undergraduate university	362	15.97
Origin	Urban Origin	1,204	53.11
	Rural Origin	1,063	46.89
First-generation status	First-Generation College Student	1,342	59.20
	Non-First-Generation College Student	925	40.80

Percentages were calculated from the baseline analytic sample. Non-Double First-Class universities included other public undergraduate universities and private undergraduate universities. Percentages may not sum to 100.00 because of rounding.

The baseline sample size was considered adequate for the planned four-wave bivariate random-intercept cross-lagged panel model (RI-CLPM). The RI-CLPM was selected because it separates stable between-person differences from within-person temporal deviations, thereby avoiding the interpretive limitation of traditional cross-lagged panel models that conflate trait-like individual differences with within-person change processes (Hamaker et al., 2015). The target sample size was set at a minimum of 2,000 participants because the expected within-person cross-lagged effects were small, the study included four repeated waves, and some attrition was anticipated across the academic year. Sample-size planning was guided by Monte Carlo principles for RI-CLPMs and by the powRICLPM framework, which was specifically developed for power analysis in random-intercept cross-lagged panel models (Mulder, 2023). The retained baseline sample of 2,267 therefore provided a large and sufficiently precise sample for estimating the planned four-wave bivariate RI-CLPM and for evaluating small but theoretically meaningful within-person cross-lagged effects (Mulder and Hamaker, 2021; Orth et al., 2024).

Attrition was carefully examined across the four waves. Among the 2,267 baseline participants, 1,801 completed all four waves, yielding a four-wave completion rate of 79.44%. A total of 2,211 participants completed at least two waves, corresponding to 97.53% of the baseline sample. With respect to missing-data patterns, 185 participants showed intermittent missingness, 272 showed sustained dropout, and 9 showed intermittent missingness followed by dropout; these groups accounted for 8.16, 12.00, and 0.40% of the baseline sample, respectively. Wave-level retention remained high throughout the study: 2,267 participants completed T1, 2,120 completed T2, 2,053 completed T3, and 1,986 completed T4, corresponding to retention rates of 100.00, 93.52, 90.56, and 87.60%, respectively.

Attrition diagnostics indicated that missingness was not completely random. Compared with participants who completed all four waves, those with incomplete follow-up reported slightly higher baseline environmental career exploration (completers: M = 3.36; non-completers: M = 3.59; standardized mean difference = 0.23, *p* < 0.001) and slightly higher baseline perceived employment outlook (completers: M = 3.18; non-completers: M = 3.38; standardized mean difference = 0.19, *p* < 0.001). The difference in baseline environmental career exploration was more pronounced among participants showing sustained dropout, with a standardized mean difference of 0.28. Additional attrition-diagnostic logistic regressions also showed that later missingness was associated with several observed baseline characteristics and prior-wave study variables. Specifically, T2 missingness was predicted by gender (OR = 1.38, *p* < 0.001). T3 missingness was predicted by gender, university tier, and T1 perceived employment outlook. T4 missingness was predicted by major, university tier, origin, first-generation status, T1 environmental career exploration, and T3 perceived employment outlook. Thus, attrition was not completely random; rather, missingness was mildly to moderately systematic and primarily related to observed demographic and study variables.

Because attrition was related to observed characteristics and prior study scores, the primary free-lag RI-CLPM used FIML with missing-data auxiliary variables. Dummy-coded gender, academic major, university tier, institution type, urban or rural origin, and first-generation status were entered through the Mplus AUXILIARY (M) option, while ECE and EPO scores were already included as model variables. This specification retained all 2,267 baseline participants and used observed information associated with later nonparticipation under a missing-at-random framework (Graham, 2009; Newman, 2014).

### 2.2 Measurements

All focal constructs were measured at four waves using the same item wording. The questionnaires were administered in Chinese. Measures originally developed in English were translated and back-translated following standard cross-cultural procedures (Brislin, 1970). Because the study focused on short-term within-person dynamics, ECE items referred to the preceding four weeks at each assessment. Scale scores were computed by averaging the corresponding items, with higher scores indicating higher construct levels. The four-week reference window captured proximal behavior near each wave and did not represent continuous exploration throughout the full 8-, 13-, or 9-week interval.

Environmental career exploration (ECE) was defined as students’ active search for information about occupations, jobs, organizations, and labor-market opportunities. It was measured with the six-item Environmental Exploration subscale of the Career Exploration Survey (Stumpf et al., 1983), which has been used with Chinese college students (Xu et al., 2014). The time frame was adapted to the preceding four weeks. A sample item is: “During the past four weeks, I actively searched for information about jobs, organizations, or occupations related to my future career.” Responses ranged from 1 = very little to 5 = a great deal and were averaged. The scale records search frequency; it does not assess the valence, credibility, relevance, or consequences of the information obtained (Jiang et al., 2019).

Perceived employment outlook (EPO) was defined as students’ subjective judgment of the likelihood of obtaining desirable employment after graduation under current labor-market conditions. It was assessed with the three-item scale used by Hodzic et al. (2015) and later applied in a Chinese undergraduate context (Ma et al., 2024). A sample item is: “Given the current job-market situation, I think I can find an interesting job after graduation.” Responses ranged from 1 = strongly disagree to 5 = strongly agree. The three items were averaged for the observed-score models. Alternative specifications retained the items as latent indicators while allowing EPO3 intercepts to vary across waves and recomputed EPO from EPO1 and EPO2 only.

### 2.3 Data analysis

All confirmatory factor analyses and structural equation models were estimated in Mplus Version 8.3 with robust maximum likelihood (MLR). The five-category items were treated as approximately continuous given the large sample (Rhemtulla et al., 2012). Longitudinal missingness was handled with FIML; the primary RI-CLPM additionally included the demographic and institutional missing-data auxiliaries described above. Tests were two-tailed with α = 0.05.

Model fit was evaluated with χ^2^, CFI, TLI, RMSEA and its 90% confidence interval, and SRMR. CFI and TLI values of at least 0.95, RMSEA of at most 0.06, and SRMR of at most 0.08 were treated as guides rather than rigid cutoffs. AIC and BIC were reported for models estimated from the same observed-variable set. Nested models estimated with MLR were compared with the appropriate scaled chi-square difference test as well as changes in approximate fit indices (Hu and Bentler, 1999; Vrieze, 2012).

Preliminary analyses included construct-specific available n, means, standard deviations, correlations, Cronbach’s α, McDonald’s ω, and ICCs. Discriminant validity was examined by comparing a longitudinal two-factor ECE–EPO measurement model with a single-factor alternative and by calculating HTMT at each wave. These checks evaluate construct separation but cannot rule out all common-method or response-style effects (Podsakoff et al., 2024).

Longitudinal measurement invariance was tested separately for ECE and EPO using item-level configural, metric, scalar, and strict models with same-item residuals correlated across waves. Changes in CFI, RMSEA, and SRMR were evaluated using established guidelines (Chen, 2007; Putnick and Bornstein, 2016). When full scalar invariance was not supported for EPO, the EPO3 intercepts were freed across waves while EPO1 and EPO2 intercepts remained equal.

A conventional free-lag CLPM was estimated as a benchmark. The RI-CLPM then decomposed each repeated score into a stable random intercept and an occasion-specific within-person component. Random-intercept and within-person loadings were fixed at 1, the two random intercepts were allowed to correlate, and same-wave within-person components were correlated. Adjacent autoregressive and reciprocal cross-lagged paths were estimated among the within-person components (Hamaker et al., 2015; Mulder and Hamaker, 2021).

Three RI-CLPM time specifications were compared: all adjacent lagged paths free; the 8- and 9-week interval paths constrained equal while the 13-week winter-recess paths remained free; and all corresponding lagged paths constrained equal. Equality constraints were imposed on unstandardized coefficients. Because the intervals were unequal, the free-lag model served as the reference and was retained when scaled difference tests indicated that the restrictions were unsupported.

The primary free-lag model with missing-data auxiliaries was used to evaluate Hypotheses 1 and 2 and Research Question 1. STDYX estimates, standard errors, 95% confidence intervals, and *p* values were reported. RI-CLPM coefficients were interpreted as prospective within-person associations, not causal effects, because unmeasured time-varying factors could affect both constructs (Rohrer and Murayama, 2023).

Measurement sensitivity was evaluated in two ways. A multiple-indicator RI-CLPM retained all ECE and EPO items, imposed longitudinal loading constraints, and allowed EPO3 intercepts to vary across waves. A second model excluded EPO3 and used the mean of EPO1 and EPO2; Spearman–Brown reliability was reported for the two-item score. The primary free-lag model was also compared with an otherwise identical model without missing-data auxiliaries.

Moderation was examined through stratified free-lag RI-CLPMs for Double First-Class versus non-Double First-Class universities and for STEM versus humanities and social sciences majors. Business, economics and management and arts and design students remained in the full-sample analysis but were not included in the STEM–humanities comparison. Cross-group differences in unstandardized paths were tested directly; interval-specific tests within each direction were adjusted with the Holm procedure. A significant path in one group and a nonsignificant path in another was not treated as evidence of moderation without a significant direct difference test.

## 3 Results

### 3.1 Descriptive statistics, reliability, and intraclass correlations

Table 2 presents construct-specific available n, descriptive statistics, reliability estimates, ICCs, and correlations. ECE means were relatively stable across waves, ranging from 3.38 to 3.49. Raw EPO composite means were higher at later waves, from 3.22 at T1 to 3.58 at T4, but this pattern is reported descriptively and is not interpreted as latent mean growth because full scalar invariance was not supported.

**Table 2:** Descriptive statistics, reliability estimates, intraclass correlations, and zero-order correlations for the study variables.

No.	Variable	*n*	M	SD	α	ω	ICC	1	2	3	4	5	6	7	8
1	ECE at T1	2,265	3.41	1.01	0.876	0.877	0.388	—							
2	ECE at T2	2,117	3.49	0.95	0.855	0.855	0.388	0.419	—						
3	ECE at T3	2,051	3.38	0.99	0.872	0.872	0.388	0.367	0.436	—					
4	ECE at T4	1,981	3.42	0.98	0.866	0.867	0.388	0.313	0.361	0.440	—				
5	EPO at T1	2,216	3.22	1.07	0.774	0.776	0.307	0.215	0.270	0.289	0.251	—			
6	EPO at T2	2,069	3.31	1.04	0.768	0.773	0.307	0.187	0.231	0.244	0.229	0.317	—		
7	EPO at T3	1,985	3.40	1.04	0.769	0.774	0.307	0.139	0.192	0.242	0.272	0.307	0.345	—	
8	EPO at T4	1,903	3.58	1.01	0.749	0.754	0.307	0.137	0.159	0.170	0.238	0.238	0.294	0.410	—

ECE, environmental career exploration; EPO, perceived employment outlook; T1–T4, Waves 1–4; α, Cronbach’s alpha; ω, McDonald’s omega; ICC, intraclass correlation coefficient. The n column reports construct-specific available observations. All zero-order correlations were significant at *p* < 0.001.

Both measures demonstrated satisfactory internal consistency. For ECE, Cronbach’s α ranged from 0.855 to 0.876 and McDonald’s ω from 0.855 to 0.877. For EPO, α ranged from 0.749 to 0.774 and ω from 0.754 to 0.776. ICCs were 0.388 for ECE and 0.307 for EPO, indicating meaningful stable between-person variance alongside substantial within-person fluctuation. Observed-score correlations ranged from 0.137 to 0.440, all *p* < 0.001.

### 3.2 Measurement properties and longitudinal invariance

The longitudinal two-factor measurement model showed excellent fit, χ^2^(512) = 521.216, *p* = 0.379, CFI = 1.000, TLI = 1.000, RMSEA = 0.003, 90% CI [0.000, 0.008], and SRMR = 0.015, and fitted substantially better than a single-factor alternative, χ^2^(534) = 6,358.178, *p* < 0.001, CFI = 0.797, TLI = 0.761, RMSEA = 0.069, and SRMR = 0.088. HTMT values ranged from 0.251 to 0.306 across waves. These results supported empirical separation of ECE and EPO while not excluding stable or time-varying self-report method effects. ECE subsequently met strict longitudinal measurement invariance, as shown in Table 3.

**Table 3:** Longitudinal measurement invariance tests for environmental career exploration and perceived employment outlook.

Variable	Model	χ^2^	df	*p*	CFI	TLI	RMSEA	RMSEA 90% CI	SRMR	ΔCFI	ΔRMSEA	ΔSRMR
ECE	Configural	201.551	210	0.650	1.000	1.001	0.000	[0.000, 0.008]	0.013	—	—	—
	Metric	220.569	225	0.571	1.000	1.000	0.000	[0.000, 0.008]	0.015	0.000	0.000	0.002
	Scalar	319.826	240	< 0.001	0.996	0.996	0.012	[0.008, 0.015]	0.018	−0.004	0.012	0.003
	Strict	364.136	258	< 0.001	0.995	0.994	0.013	[0.010, 0.017]	0.020	−0.001	0.001	0.002
EPO	Configural	35.439	30	0.227	0.999	0.998	0.009	[0.000, 0.019]	0.012	—	—	—
	Metric	45.281	36	0.138	0.999	0.998	0.011	[0.000, 0.019]	0.017	0.000	0.002	0.005
	Full scalar	159.912	42	< 0.001	0.984	0.974	0.035	[0.030, 0.041]	0.029	−0.015	0.024	0.012
	Partial scalar	56.454	39	0.035	0.998	0.996	0.014	[0.004, 0.022]	0.017	−0.001	0.003	0.000

ECE, environmental career exploration; EPO, perceived employment outlook; CFI, comparative fit index; TLI, Tucker–Lewis index; RMSEA, root mean square error of approximation; CI, confidence interval; SRMR, standardized root mean square residual. Changes in fit indices were calculated relative to the immediately preceding, less constrained model, except for the EPO partial scalar model, for which ΔCFI, ΔRMSEA, and ΔSRMR were calculated relative to the EPO metric model. In the EPO partial scalar model, the intercepts of the third EPO item were freely estimated across waves, whereas the intercepts of the first two EPO items remained constrained to equality. A full strict invariance model for EPO was not retained as a primary measurement model because full scalar invariance was not supported.

For EPO, configural and metric invariance were supported, but full scalar invariance was not. The full scalar model fitted acceptably in absolute terms, χ^2^(42) = 159.912, CFI = 0.984, TLI = 0.974, RMSEA = 0.035, and SRMR = 0.029, but change indices exceeded the prespecified criteria. Freeing the EPO3 intercepts across waves while retaining equality for EPO1 and EPO2 produced excellent fit, χ^2^(39) = 56.454, CFI = 0.998, TLI = 0.996, RMSEA = 0.014, and SRMR = 0.017. EPO raw mean differences were therefore not interpreted as latent mean change. The multiple-indicator sensitivity model explicitly retained this partial-invariance specification.

### 3.3 Temporal model comparison and primary model

A conventional CLPM with freely estimated adjacent paths showed inadequate fit, χ^2^ (12) = 380.538, *p* < 0.001, CFI = 0.864, TLI = 0.694, RMSEA = 0.116, and SRMR = 0.072. The corresponding free-lag RI-CLPM fitted well, χ^2^ (9) = 48.092, *p* < 0.001, CFI = 0.986, TLI = 0.957, RMSEA = 0.044, and SRMR = 0.030, supporting separation of stable between-person differences from within-person temporal deviations (Table 4).

**Table 4:** Fit indices for the conventional cross-lagged panel model and alternative random-intercept cross-lagged panel models.

Model	χ^2^ (df)	*p*	CFI	TLI	SRMR	RMSEA [90% CI]	ΔCFI	ΔRMSEA	ΔSRMR	AIC	BIC	Role
CLPM, free lags	380.538 (12)	< 0.001	0.864	0.694	0.072	0.116 [0.106, 0.127]	—	—	—	44,906.96	45,090.20	Benchmark
RI-CLPM, free lags	48.092 (9)	< 0.001	0.986	0.957	0.030	0.044 [0.032, 0.056]	—	—	—	44,576.89	44,777.31	Reference
RI-CLPM, short intervals equal	67.227 (13)	< 0.001	0.981	0.958	0.028	0.043 [0.033, 0.053]	−0.005	−0.001	−0.002	44,588.14	44,765.65	Not retained
RI-CLPM, equal lags	69.234 (17)	< 0.001	0.981	0.969	0.028	0.037 [0.028, 0.046]	−0.005	−0.007	−0.002	44,582.49	44,737.10	Not retained
RI-CLPM, free lags with auxiliaries	48.333 (9)	< 0.001	0.986	0.956	0.030	0.044 [0.032, 0.056]	—	—	—	—	—	Primary

CLPM, cross-lagged panel model; RI-CLPM, random-intercept cross-lagged panel model; CFI, comparative fit index; TLI, Tucker–Lewis index; SRMR, standardized root mean square residual; RMSEA, root mean square error of approximation; CI, confidence interval; AIC, Akaike information criterion; BIC, Bayesian information criterion. In the short-interval-equal model, corresponding paths across T1 → T2 and T3 → T4 were constrained equal while T2 → T3 paths remained free. The equal-lag model constrained all corresponding lagged paths. Changes in approximate fit indices are relative to the nonauxiliary free-lag RI-CLPM. MLR-scaled difference tests were significant for the short-interval-equal model, Δχ^2^(4) = 19.200, *p* < 0.001, and the equal-lag model, Δχ^2^(8) = 21.597, *p* = 0.006. AIC favored the free-lag model, whereas BIC favored the equal-lag model. AIC and BIC are not shown for the auxiliary-variable model because its likelihood incorporated additional observed variables.

The model constraining corresponding paths across the two shorter intervals while leaving the 13-week winter-recess interval free also fitted acceptably, χ^2^ (13) = 67.227, *p* < 0.001, CFI = 0.981, TLI = 0.958, RMSEA = 0.043, and SRMR = 0.028. However, the MLR-scaled comparison with the free-lag model was significant, Δχ^2^ (4) = 19.200, *p* < 0.001. The fully equal-lag model fitted well in absolute terms, χ^2^ (17) = 69.234, *p* < 0.001, CFI = 0.981, TLI = 0.969, RMSEA = 0.037, and SRMR = 0.028, but its scaled comparison with the free-lag model was also significant, Δχ^2^ (8) = 21.597, *p* = 0.006.

AIC favored the free-lag RI-CLPM, whereas BIC favored the fully constrained model. Given the significant scaled difference tests, the unequal 8-, 13-, and 9-week intervals, and the distinct winter-recess context, the free-lag specification was retained. The primary model included missing-data auxiliaries and showed nearly identical fit, χ^2^ (9) = 48.333, *p* < 0.001, CFI = 0.986, TLI = 0.956, RMSEA = 0.044, 90% CI [0.032, 0.056], and SRMR = 0.030. AIC and BIC were not compared between auxiliary and nonauxiliary models because their likelihoods incorporated different observed-variable sets.

### 3.4 Between-person and interval-specific within-person associations

**Figure 2:**
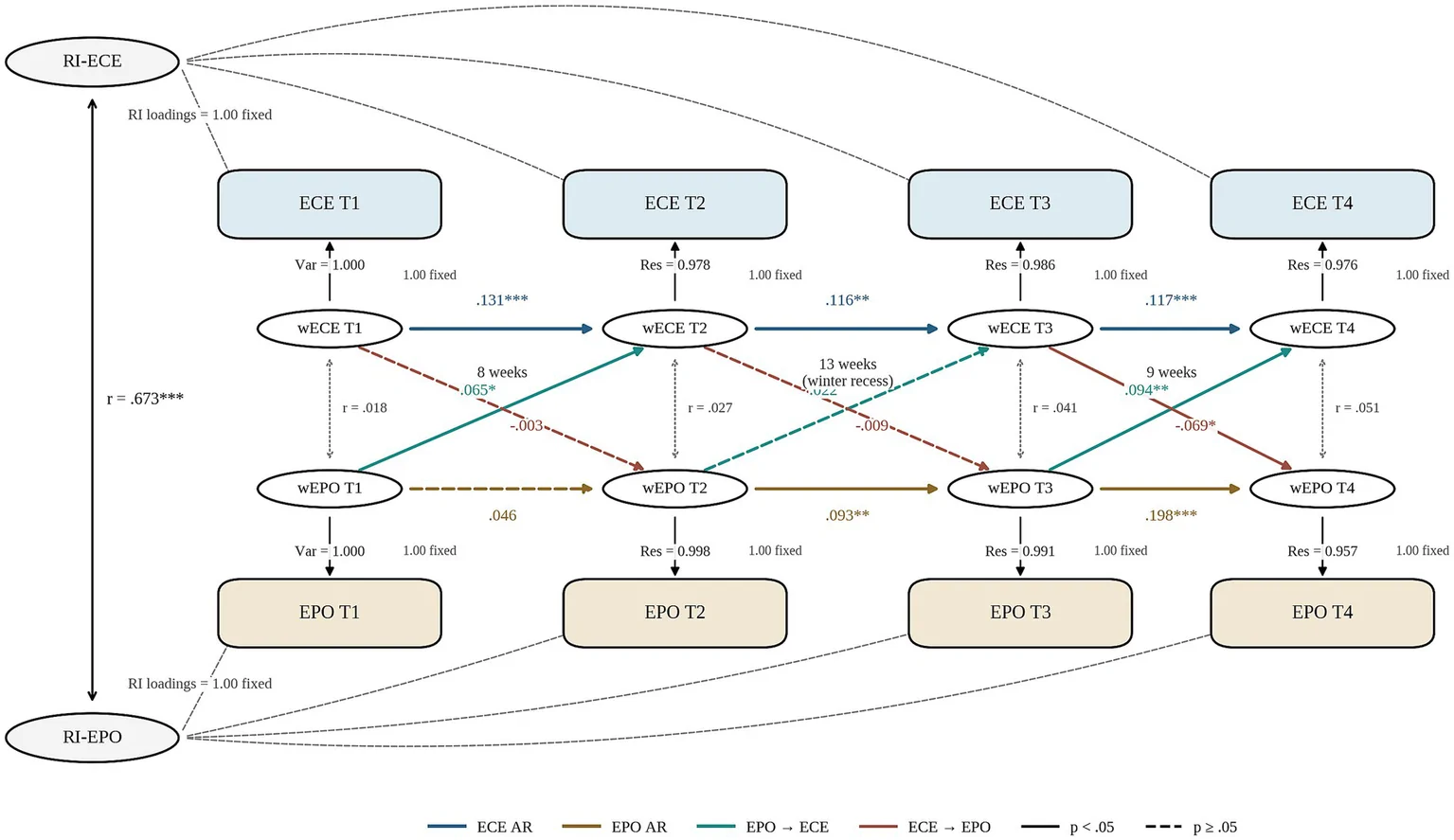
Standardized estimates from the primary free-lag RI-CLPM with missing-data auxiliaries. ECE, environmental career exploration; EPO, perceived employment outlook; RI, random intercept; *w*, within-person deviation; T1–T4, Waves 1–4. Random-intercept and within-person loadings were fixed at 1. Solid directional paths are significant at *p* < 0.05; dashed paths are nonsignificant. Values are STDYX standardized estimates. Variance values beside *w*ECE and *w*EPO are standardized variances at T1 and residual variances at T2–T4. *p < 0.05; **p < 0.01; ***p < 0.001.

**Figure 3:**
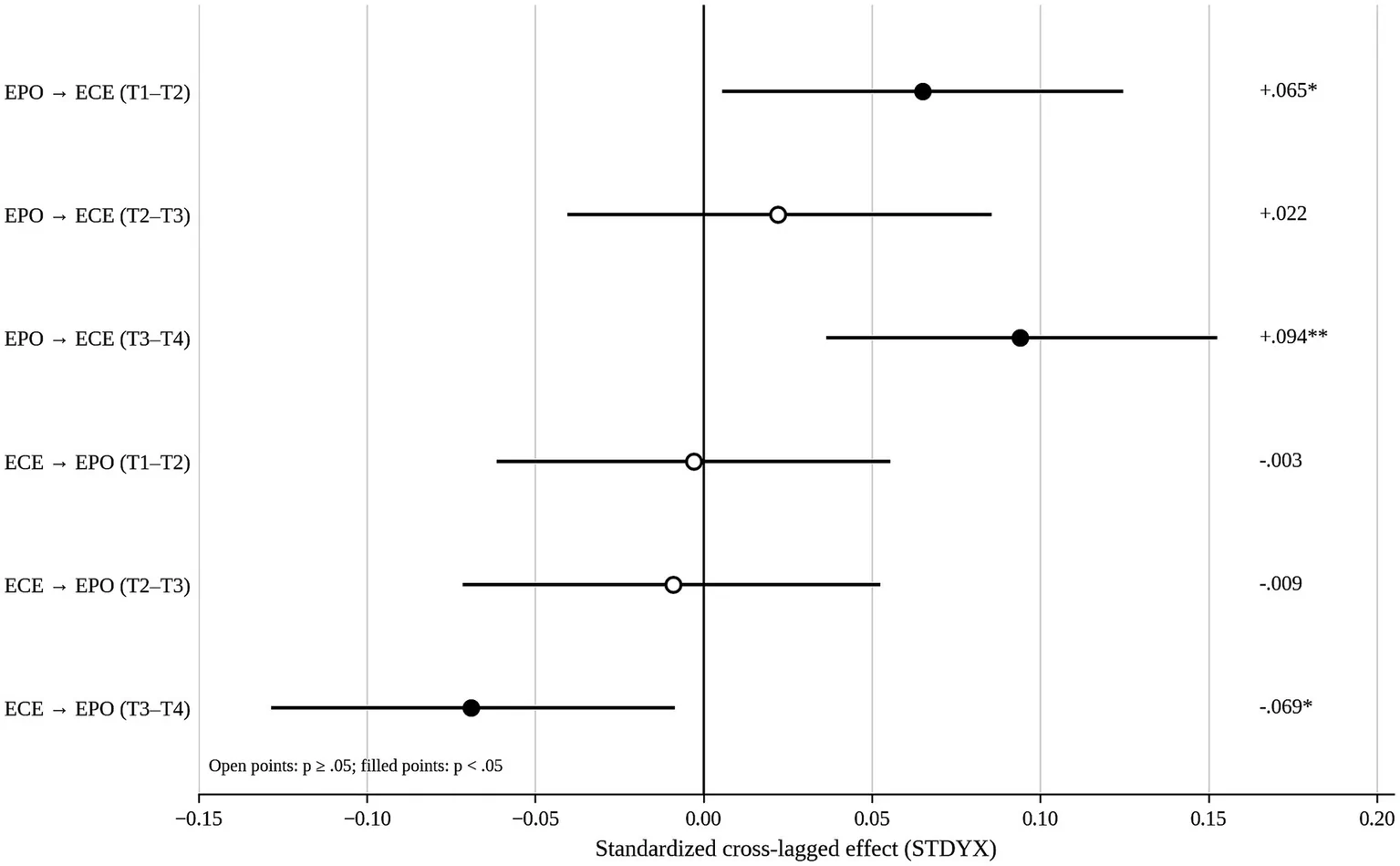
Forest plot of interval-specific standardized cross-lagged effects from the primary free-lag RI-CLPM. ECE, environmental career exploration; EPO, perceived employment outlook; T1–T4, Waves 1–4. Points are STDYX estimates and horizontal lines are 95% confidence intervals. Filled points indicate *p* < 0.05; open points indicate *p* ≥ 0.05. The vertical reference line is zero.

Standardized estimates from the primary free-lag RI-CLPM with missing-data auxiliaries are reported in Table 5 and Figures 2, 3. The random intercepts of ECE and EPO were strongly and positively correlated, r = 0.673, 95% CI [0.594, 0.751], *p* < 0.001. Students with generally more favorable employment outlooks therefore also tended to report more environmental career exploration across the study period.

**Table 5:** Parameter estimates from the primary free-lag random-intercept cross-lagged panel model with missing-data auxiliaries.

Path	Interval	b	SE	β or r	Standardized SE	95% CI for β or r	*p*
Between-person association							
RI-ECE ↔ RI-EPO	Between-person	0.211	0.015	0.673	0.040	[0.594, 0.751]	< 0.001
Within-person autoregressive effects							
ECE T1 → ECE T2	T1 → T2	0.119	0.029	0.131	0.031	[0.070, 0.191]	< 0.001
ECE T2 → ECE T3	T2 → T3	0.119	0.035	0.116	0.034	[0.049, 0.183]	0.001
ECE T3 → ECE T4	T3 → T4	0.117	0.031	0.117	0.031	[0.056, 0.178]	< 0.001
EPO T1 → EPO T2	T1 → T2	0.044	0.031	0.046	0.032	[−0.017, 0.108]	0.151
EPO T2 → EPO T3	T2 → T3	0.094	0.034	0.093	0.034	[0.028, 0.159]	0.005
EPO T3 → EPO T4	T3 → T4	0.195	0.031	0.198	0.031	[0.138, 0.258]	< 0.001
Cross-lagged effects: EPO predicting ECE							
EPO T1 → ECE T2	T1 → T2	0.055	0.026	0.065	0.030	[0.006, 0.124]	0.030
EPO T2 → ECE T3	T2 → T3	0.020	0.029	0.022	0.032	[−0.040, 0.085]	0.482
EPO T3 → ECE T4	T3 → T4	0.084	0.026	0.094	0.029	[0.037, 0.152]	0.001
Cross-lagged effects: ECE predicting EPO							
ECE T1 → EPO T2	T1 → T2	−0.003	0.031	−0.003	0.029	[−0.061, 0.055]	0.914
ECE T2 → EPO T3	T2 → T3	−0.011	0.036	−0.009	0.031	[−0.071, 0.052]	0.765
ECE T3 → EPO T4	T3 → T4	−0.076	0.034	−0.069	0.030	[−0.128, −0.009]	0.024

ECE, environmental career exploration; EPO, perceived employment outlook; RI, random intercept; T1–T4, Waves 1–4; b, unstandardized estimate; SE, standard error of the unstandardized estimate; β, STDYX standardized regression coefficient; r, standardized correlation; CI, confidence interval. All adjacent lagged parameters were freely estimated.

Within-person autoregressive estimates varied across intervals. ECE showed modest carryover from T1 to T2, β = 0.131, 95% CI [0.070, 0.191], *p* < 0.001; T2 to T3, β = 0.116, 95% CI [0.049, 0.183], *p* = 0.001; and T3 to T4, β = 0.117, 95% CI [0.056, 0.178], *p* < 0.001. EPO carryover was nonsignificant from T1 to T2, β = 0.046, 95% CI [−0.017, 0.108], *p* = 0.151, and significant from T2 to T3, β = 0.093, 95% CI [0.028, 0.159], *p* = 0.005, and T3 to T4, β = 0.198, 95% CI [0.138, 0.258], *p* < 0.001.

Higher-than-usual EPO predicted higher subsequent ECE from T1 to T2, β = 0.065, 95% CI [0.006, 0.124], *p* = 0.030, and from T3 to T4, β = 0.094, 95% CI [0.037, 0.152], *p* = 0.001. The corresponding association across the longer winter-recess interval from T2 to T3 was smaller and nonsignificant, β = 0.022, 95% CI [−0.040, 0.085], *p* = 0.482. Hypothesis 2 was therefore supported in two of the three adjacent intervals rather than uniformly across the academic year.

Higher-than-usual ECE was unrelated to subsequent EPO from T1 to T2, β = −0.003, 95% CI [−0.061, 0.055], *p* = 0.914, and from T2 to T3, β = −0.009, 95% CI [−0.071, 0.052], *p* = 0.765. From T3 to T4, higher-than-usual ECE predicted a modest decline in subsequent EPO, β = −0.069, 95% CI [−0.128, −0.009], *p* = 0.024. Same-wave within-person correlations ranged from r = 0.018 to 0.051 and were nonsignificant.

The prospective pattern was interval specific. Positive EPO-to-ECE associations appeared before the winter recess and again in late spring but were not detected across the winter-recess interval. A negative ECE-to-EPO association appeared only in the final interval. The data therefore did not support a temporally uniform one-way or reciprocal process.

### 3.5 Measurement, missing-data, and group sensitivity analyses

The multiple-indicator RI-CLPM, which retained all items and allowed EPO3 intercepts to vary across waves, fitted well, χ^2^(566) = 805.830, *p* < 0.001, CFI = 0.992, TLI = 0.991, RMSEA = 0.014, and SRMR = 0.028. It reproduced the primary interval-specific pattern: EPO predicted ECE from T1 to T2, β = 0.083, *p* = 0.049, and T3 to T4, β = 0.104, *p* = 0.008, but not T2 to T3, β = 0.045, *p* = 0.310; ECE predicted lower EPO only from T3 to T4, β = −0.122, *p* = 0.002. The between-person correlation was r = 0.683, *p* < 0.001 (Supplementary Table S1).

A two-item EPO score excluding EPO3 also produced the same pattern. Spearman–Brown reliability was 0.755, 0.760, 0.765, and 0.733 across the four waves. In the free-lag model with auxiliaries, EPO-to-ECE estimates were β = 0.070, *p* = 0.016; β = 0.025, *p* = 0.432; and β = 0.072, *p* = 0.015. ECE-to-EPO estimates were β = 0.005, *p* = 0.858; β = −0.011, *p* = 0.734; and β = −0.072, *p* = 0.016. Thus, the final-interval negative association and the absence of the middle-interval EPO-to-ECE association did not depend on EPO3.

Including missing-data auxiliaries changed the focal standardized coefficients by no more than 0.001 relative to the otherwise identical free-lag model without auxiliaries, and no significance conclusion changed. The similarity indicates that use of observed attrition predictors improved the missing-data specification without materially altering the estimated focal associations (Supplementary Table S1).

Stratified analyses provided no multiplicity-adjusted evidence that the cross-lagged paths differed between Double First-Class (*n* = 342) and non-Double First-Class universities (*n* = 1,925). A nominal university-tier difference for the T3-to-T4 ECE-to-EPO path, *p* = 0.035, did not survive Holm correction, adjusted *p* = 0.104. Non-Double First-Class universities included both other public and private undergraduate institutions.

The STEM (*n* = 964) and humanities and social sciences (*n* = 558) comparisons likewise showed no cross-group difference after Holm correction. Business, economics and management and arts and design students remained in the full-sample model but were excluded from this two-group comparison. These stratified models test observed group heterogeneity, not person-specific random slopes (Supplementary Table S2).

## 4 Discussion

The present four-wave study examined prospective ECE–EPO dynamics among undergraduates enrolled at 16 participating universities in Liaoning Province. Separating stable between-person differences from within-person deviations and allowing the three intervals to differ produced a more conditional pattern than a fully equal-lag summary would imply.

ECE and EPO were strongly related at the between-person level. Within persons, however, higher-than-usual EPO preceded greater ECE in the first and final intervals but not across the winter recess, and higher-than-usual ECE preceded a modest decline in EPO only in the final interval. The pattern remained under multiple-indicator and EPO3-exclusion specifications and after incorporating missing-data auxiliaries. No university-tier or academic-major path difference survived multiplicity correction. The main contribution is therefore an interval-dependent account rather than evidence of a uniform asymmetric process.

### 4.1 Interpreting the interval-specific within-person dynamics

The contrast between the strong random-intercept correlation and the small same-wave within-person correlations remains theoretically important. Students who were characteristically more favorable about their employment prospects also tended to be characteristically more exploratory, but short-term deviations in the two constructs were only weakly synchronized. Stable career preparedness, support, information access, institutional resources, or response tendencies may contribute to the between-person association, consistent with recent evidence that employability reflects configurations of personal and contextual career resources (Antonio and Chiesa, 2024; Paganin et al., 2025). The RI-CLPM separates levels of variation but does not identify the attributes underlying the random-intercept correlation (Hamaker et al., 2015; Rohrer and Murayama, 2023).

The positive EPO-to-ECE estimates in the first and final intervals are consistent with the motivational logic of career self-management and situated expectancy-value perspectives. When suitable employment appears attainable, searching for employers, occupational routes, and job requirements may have a clearer anticipated return (Eccles and Wigfield, 2020; Lent and Brown, 2013). The estimates are prospective associations rather than evidence that EPO caused exploration, and the nonsignificant middle-interval path shows that the association was not invariant across the academic year.

EPO should not be treated as generic confidence or exploration-specific self-efficacy. It reflects a context-sensitive appraisal of employment opportunity. Its relation with exploration may arise because perceived attainability changes the anticipated usefulness of action, a process that is compatible with recent research linking future orientation, career decision confidence, self-regulation, and proactive career behavior (Badwy et al., 2025; Qalati et al., 2025). The present study did not directly measure these intervening motivational processes.

The absence of an EPO-to-ECE association across the 13-week winter-recess interval deserves attention. That interval was longer and likely differed from the two teaching-period intervals in academic demands, location, exposure to family expectations, recruitment activity, internships, and postgraduate planning. None of these time-varying factors was measured. The result therefore supports interval sensitivity but does not identify why the association weakened during that period.

The negative ECE-to-EPO association from T3 to T4 is compatible with downward expectation revision after increased contact with labor-market information. It cannot show that exploration was ineffective, harmful, or more accurately calibrated. ECE measured search frequency without identifying the information encountered, and EPO was not compared with objective job opportunities or later employment outcomes. The earlier nonsignificant paths also cannot establish that positive and negative informational experiences canceled one another.

Search quantity may have different consequences depending on information quality, credibility, relevance, and interpretation. Similar amounts of exploration could involve superficial browsing, focused occupational research, direct employer contact, or exposure to discouraging qualification requirements. Future work should measure these features directly and test whether labor-market knowledge, self-regulated learning, employability capital, or career decision processes mediate or moderate changes in EPO (Haenggli et al., 2025; Qalati et al., 2025).

The multiple-indicator model and the two-item EPO model strengthened confidence that the interval-specific pattern was not an artifact of the EPO3 intercept shift. These analyses do not erase the measurement limitation: raw EPO mean increases remain difficult to interpret, and all focal measures were self-reported. The strong improvement of the two-factor measurement model over a single-factor alternative supports construct separation, while stable and time-varying method effects may still contribute to associations.

The stratified analyses did not detect university-tier or academic-major differences after correction. These results reduce concern that the full-sample pattern was driven entirely by the examined coarse groupings, but they do not establish homogeneity across institutions, disciplines, or individuals. The Double First-Class group was comparatively small, the major comparison excluded business and arts students, and the available data did not contain a recoverable institution identifier for evaluating school-level clustering.

Taken together, the results support a temporally and contextually qualified account. EPO sometimes preceded greater exploration, but the association was absent across one interval. Exploration sometimes preceded downward revision in EPO, but only late in the academic year. Career-development theory should therefore distinguish stable between-person alignment, interval-specific average dynamics, and unobserved heterogeneity rather than describing ECE and EPO as a single uniform feedback process.

### 4.2 Theoretical implications

The findings reinforce the importance of specifying the level and timing of career processes. A strong between-person association can coexist with weak or changing within-person paths. Theories that do not distinguish enduring differences from occasion-specific deviations may attribute a developmental mechanism to an association sustained by stable personal, institutional, contextual, or method-related factors (Hamaker et al., 2015; Usami, 2021).

EPO may function as a proximal appraisal of whether career action is worth pursuing. This extends career self-management perspectives by emphasizing perceived opportunity alongside perceived capability. The first- and final-interval EPO-to-ECE paths are consistent with that account, while their absence across the winter recess shows that the motivational relevance of EPO is conditional rather than temporally uniform.

The reverse pathway has a less determinate direction because ECE measures behavior frequency while EPO reflects an evaluative conclusion. Exploration must expose students to information before that information can be interpreted, incorporated into knowledge, and used to revise expectations. Recent research on career proactivity, self-regulated learning, future orientation, and career decision confidence supports theorizing these intermediate processes explicitly (Badwy et al., 2025; Qalati et al., 2025). The present estimates do not demonstrate any particular mediation mechanism.

Opportunity discovery and constraint discovery remain plausible informational pathways, but they should be treated as testable alternatives. A positive path would be compatible with discovery of attainable options, whereas a negative path would be compatible with downward revision after encountering constraints. A nonsignificant coefficient cannot reveal whether the processes were absent, balanced, heterogeneous, or poorly timed. Future models should assess information valence, credibility, labor-market knowledge, and resource acquisition directly.

The regional and institutional setting also belongs within the theoretical boundary. Liaoning employment policy places emphasis on supply–demand matching, program adjustment, skills conversion, and career services, illustrating how career appraisals and behavior are embedded in educational and labor-market systems. Contemporary employability research likewise emphasizes that development unfolds within contextual opportunities, barriers, and institutional conditions (Akkermans et al., 2024; Rodrigues et al., 2024). Related evidence on human-resource practices and career sustainability similarly highlights the role of contextual resources and psychological states, although it comes from an employee hospitality setting rather than undergraduate career exploration (Qalati et al., 2026). The present study did not measure policy exposure or intended employment location, so the regional context limits generalization rather than serving as an identified explanatory variable.

### 4.3 Practical implications

The findings caution against evaluating career exploration solely by whether students become immediately more optimistic. Greater ECE preceded lower EPO in the final interval, but the study did not assess whether the change reflected more realistic knowledge, discouraging information, or an unmeasured event. Career programs should evaluate information quality, occupational knowledge, decision clarity, and actionable next steps alongside EPO.

Students reporting less favorable employment outlooks may also be less likely to engage in exploration during some periods. Brief, nonstigmatizing assessments could help career services identify students who may benefit from proactive invitations to structured activities. Whether an intervention that changes EPO subsequently increases ECE requires experimental evaluation; the observational paths do not justify a causal intervention claim.

Structured exploration can combine employer contact, qualification mapping, labor-market interpretation, transferable-skill identification, and written action planning. Such design places emphasis on what students learn and how they use the information rather than on attendance or search counts alone. Recent evidence links work experience and active career engagement with students’ employability-related learning and appraisals (Brosnan et al., 2024; Tuononen et al., 2024). Counselor support and opportunities to process information may be especially valuable when students encounter barriers or revise prior expectations (Brown et al., 2003; Whiston et al., 2017).

The absence of corrected university-tier and major differences does not support separate causal prescriptions for the examined groups. Career services can nevertheless align information with regional industries, institutional resources, and discipline-specific routes while monitoring whether support reaches students whose perceived opportunities are limited. Claims should remain bounded to participating Liaoning universities and comparable settings.

Repeated assessment may be more informative than a single annual evaluation because the paths varied across the academic year. Short check-ins before and after recruitment periods, internships, examinations, and postgraduate decision points could help practitioners observe changes in EPO and ECE. Such monitoring should support individualized guidance without treating either construct as fixed or assuming that one necessarily changes the other.

### 4.4 Limitations and future research

Several limitations qualify the findings. The RI-CLPM separated stable between-person differences from within-person deviations but did not establish causality. Internships, recruitment events, career counseling, academic demands, postgraduate plans, family discussions, and labor-market information could have influenced both EPO and ECE. Experimental designs, randomized encouragement, or longitudinal studies with measured time-varying covariates are needed to identify causal effects (Muthén and Asparouhov, 2024; Rohrer and Murayama, 2023).

Both constructs were self-reported. The longitudinal two-factor model and low HTMT values supported empirical distinction between ECE and EPO, but these checks cannot remove stable response styles, mood-congruent reporting, or other common-method influences. Behavioral indicators such as career-platform activity, employer-event attendance, informational interviews, and applications would provide useful multimethod evidence (Podsakoff et al., 2024).

ECE assessed the frequency of external information seeking, not its source, quality, valence, credibility, or consequences. The data therefore cannot determine whether students discovered opportunities, encountered constraints, or translated information into knowledge and action. Diary or event-contingent designs could measure individual informational episodes and test these mechanisms directly.

EPO was measured with three items and achieved partial rather than full scalar invariance because EPO3 intercepts differed across waves. Raw mean changes should therefore remain descriptive. The multiple-indicator model with freely estimated EPO3 intercepts and the EPO1–EPO2 sensitivity model reproduced the principal interval-specific findings, reducing but not eliminating concern about measurement specification.

The adjacent intervals were approximately 8, 13, and 9 weeks, and the middle interval crossed the winter recess. Free estimation addressed unsupported equality constraints, but cross-lagged coefficients remain dependent on the selected lag. The four-week ECE reference window also captured only the proximal period before each survey rather than behavior throughout each complete interval. More frequent assessments and exact dates would permit analysis across finer temporal scales (Dormann and Griffin, 2015; Driver, 2025).

The RI-CLPM estimated average within-person paths. Stratified models did not detect corrected differences by university tier or between STEM and humanities and social sciences, but these categories are coarse and cannot represent person-specific dynamics. More repeated observations would be required for random-slope or dynamic models capable of estimating individual heterogeneity.

Participants came from 16 universities in Liaoning Province, including Double First-Class, other public, and private undergraduate institutions. The sample did not cover vocational institutions, other provinces, or all regional labor markets and cannot represent Chinese undergraduates generally. Students’ intended employment locations were not measured, so their EPO may have referred to provincial, hometown, national, or other labor markets. Replication across regions, institution types, disciplines, and transition stages is needed.

Attrition was associated with observed demographic characteristics and prior scores. Incorporating these predictors as FIML auxiliaries produced estimates nearly identical to the nonauxiliary model, but the missing-at-random assumption remains untestable and unmeasured states may have influenced participation. No recoverable institution identifier was available, preventing evaluation of school-level clustering despite recruitment from 16 universities.

EPO was not compared with objective employment outcomes. An increase or decrease cannot therefore be interpreted as better calibration. Following students through graduation and linking EPO with applications, interviews, offers, employment timing, job–education match, and job quality would clarify whether exploration changes the accuracy, positivity, or both dimensions of employment expectations.

## 5 Conclusion

Among undergraduates at participating universities in Liaoning Province, ECE and EPO were strongly related as stable between-person tendencies. Their within-person prospective associations were interval dependent: higher-than-usual EPO preceded higher ECE before the winter recess and again in late spring, whereas higher-than-usual ECE preceded a modest downward shift in EPO only in the final interval. These associations remained under item-level and EPO3-exclusion specifications, but the observational design does not establish causality. Because ECE captured search frequency rather than information content, the final negative path cannot show that exploration is ineffective or identify whether students encountered opportunities, constraints, or both. Career support should therefore assess the quality and interpretation of exploration alongside students’ employment outlook while recognizing the regional limits of the evidence.

## Data Availability

The original contributions presented in the study are included in the article/Supplementary material, further inquiries can be directed to the corresponding author.

## References

[ref1] Akkermans J. Le Blanc P. M. van der Heijden B. I. J. M. De Vos A. (2024). Toward a contextualized perspective of employability development. Eur. J. Work Organ. Psychol. 33, 1–10. doi: 10.1080/1359432X.2023.2291763

[ref2] Ananthram S. Bawa S. Bennett D. Gill C. (2024). Perceived employability and career readiness among STEM students: does gender matter? High. Educ. Res. Dev. 43, 267–283. doi: 10.1080/07294360.2023.2240710

[ref3] Antonio A. A. Chiesa R. (2024). Exploring university students’ career resources profiles to cope with career insecurity and promote employability. Soc. Sci. 13:Article 455. doi: 10.3390/socsci13090455

[ref4] Ayvaz A. Elhatip Y. E. (2026). From perseverance of effort to perceived employability in first-generation college students: the mediating role of career adaptability and career engagement. J. Career Assess. 34, 62–79. doi: 10.1177/10690727251313789

[ref5] Azalea A. Lim P. K. Lin M.-H. Lee M. C. C. (2026). Effects of career adaptability on university fresh graduates’ perceived future employability and career motivation: employed versus unemployed. Int. J. Educ. Vocat. Guid. 26, 231–258. doi: 10.1007/s10775-024-09716-0

[ref6] Badwy H. E. Qalati S. A. El-Bardan M. F. (2025). Unlocking happiness: how crafting your career path boosts life satisfaction through forward thinking and self-confidence in career choices. Hum. Soc. Sci. Commun. 12:384. doi: 10.1057/s41599-025-04655-9

[ref7] Brislin R. W. (1970). Back-translation for cross-cultural research. J. Cross-Cult. Psychol. 1, 185–216. doi: 10.1177/135910457000100301

[ref8] Brosnan M. Bennett D. Kercher K. Wilson T. Keogh J. W. L. (2024). A multi-institution study of the impacts of concurrent work and study among university students in Australia. High. Educ. Res. Dev. 43, 775–791. doi: 10.1080/07294360.2023.2287722

[ref9] Brown S. D. Ryan Krane N. E. Brecheisen J. Castelino P. Budisin I. Miller M. . (2003). Critical ingredients of career choice interventions: more analyses and new hypotheses. J. Vocat. Behav. 62, 411–428. doi: 10.1016/S0001-8791(02)00052-0

[ref10] Chen F. F. (2007). Sensitivity of goodness of fit indexes to lack of measurement invariance. Struct. Equ. Model. 14, 464–504. doi: 10.1080/10705510701301834

[ref11] Chen H. Wu Y. Jiang L. Xu B. Gao X. Cai W. (2023). Future orientation and perceived employability of Chinese undergraduates: a moderated mediation model. Curr. Psychol. 42, 27127–27140. doi: 10.1007/s12144-022-03769-6, 36254214 PMC9556284

[ref12] Cole D. A. Maxwell S. E. (2003). Testing mediational models with longitudinal data: questions and tips in the use of structural equation modeling. J. Abnorm. Psychol. 112, 558–577. doi: 10.1037/0021-843X.112.4.558 14674869

[ref13] Dormann C. Griffin M. A. (2015). Optimal time lags in panel studies. Psychol. Methods 20, 489–505. doi: 10.1037/met0000041, 26322999

[ref14] Driver C. C. (2025). Inference with cross-lagged effects—problems in time. Psychol. Methods 30, 174–202. doi: 10.1037/met0000665 39023978

[ref15] Eccles J. S. Wigfield A. (2020). From expectancy-value theory to situated expectancy-value theory: a developmental, social cognitive, and sociocultural perspective on motivation. Contemp. Educ. Psychol. 61:Article 101859. doi: 10.1016/j.cedpsych.2020.101859

[ref16] Flum H. Blustein D. L. (2000). Reinvigorating the study of vocational exploration: a framework for research. J. Vocat. Behav. 56, 380–404. doi: 10.1006/jvbe.2000.1721

[ref17] Gerçek M. (2024). Serial multiple mediation of career adaptability and self-perceived employability in the relationship between career competencies and job search self-efficacy. High. Educ. Skills Work-Based Learn. 14, 461–478. doi: 10.1108/HESWBL-02-2023-0036

[ref18] Graham J. W. (2009). Missing data analysis: making it work in the real world. Annu. Rev. Psychol. 60, 549–576. doi: 10.1146/annurev.psych.58.110405.085530 18652544

[ref19] Grosemans I. Forrier A. De Cuyper N. (2024). Dynamic interconnections between career engagement and perceived employability among recent graduates: a latent change score modeling approach. Higher Educ. Skills Work-Based Learn. 14, 850–864. doi: 10.1108/HESWBL-03-2024-0072

[ref20] Haenggli M. Hirschi A. Marciniak J. (2025). Navigating transitions: a longitudinal exploration of career decision-making process dynamics in adolescents. J. Vocat. Behav. 159:104125. doi: 10.1016/j.jvb.2025.104125, 42574925

[ref21] Hamaker E. L. Kuiper R. M. Grasman R. P. P. P. (2015). A critique of the cross-lagged panel model. Psychol. Methods 20, 102–116. doi: 10.1037/a0038889, 25822208

[ref22] Hodzic S. Ripoll P. Lira E. Zenasni F. (2015). Can intervention in emotional competences increase employability prospects of unemployed adults? J. Vocat. Behav. 88, 28–37. doi: 10.1016/j.jvb.2015.02.007

[ref23] Hu L.-T. Bentler P. M. (1999). Cutoff criteria for fit indexes in covariance structure analysis: conventional criteria versus new alternatives. Struct. Equ. Model. Multidiscip. J. 6, 1–55. doi: 10.1080/10705519909540118

[ref24] Huang J. L. Curran P. G. Keeney J. Poposki E. M. DeShon R. P. (2012). Detecting and deterring insufficient effort responding to surveys. J. Bus. Psychol. 27, 99–114. doi: 10.1007/s10869-011-9231-8

[ref25] Jiang Z. Newman A. Le H. Presbitero A. Zheng C. (2019). Career exploration: a review and future research agenda. J. Vocat. Behav. 110, 338–356. doi: 10.1016/j.jvb.2018.08.008

[ref26] Kirazci F. (2026). Can I still dream of a decent work? The interplay of economic constraints, perceived employability, and social support. Int. J. Educ. Vocat. Guid. doi: 10.1007/s10775-025-09789-5

[ref27] Kleine A.-K. Schmitt A. Wisse B. (2021). Students’ career exploration: a meta-analysis. J. Vocat. Behav. 131:103645. doi: 10.1016/j.jvb.2021.103645, 42574925

[ref28] Lent R. W. Brown S. D. (2013). Social cognitive model of career self-management: toward a unifying view of adaptive career behavior across the life span. J. Couns. Psychol. 60, 557–568. doi: 10.1037/a0033446, 23815631

[ref29] Lent R. W. Ezeofor I. Morrison M. A. Penn L. T. Ireland G. W. (2016). Applying the social cognitive model of career self-management to career exploration and decision-making. J. Vocat. Behav. 93, 47–57. doi: 10.1016/j.jvb.2015.12.007

[ref30] Lent R. W. Morris T. R. Penn L. T. Ireland G. W. (2019). Social-cognitive predictors of career exploration and decision-making: longitudinal test of the career self-management model. J. Couns. Psychol. 66, 184–194. doi: 10.1037/cou0000307, 30091621

[ref31] Liaoning Provincial Department of Education (2025) Liaoning Province convenes employment and entrepreneurship work conference for the 2026 graduating class. Available online at: https://jyt.ln.gov.cn/jyt/jyzx/jyyw/2025122516391218543/index.shtml (accessed August 30, 2026).

[ref32] Liaoning Provincial Department of Human Resources and Social Security (2025) Notice on employment and entrepreneurship for 2025 college graduates and other young people. Available online at: https://www.ln.gov.cn/web/zwgkx/lnsrmzfgb/2025n/qk/2025n_dslq/bmwj/2025081409115090546/index.shtml (accessed August 30, 2026).

[ref33] Ma Y. Hou L. Cai W. Gao X. Jiang L. (2024). Linking undergraduates’ future work self and employability: a moderated mediation model. BMC Psychol. 12:Article 160. doi: 10.1186/s40359-024-01530-1, 38500193 PMC10949635

[ref34] Meade A. W. Craig S. B. (2012). Identifying careless responses in survey data. Psychol. Methods 17, 437–455. doi: 10.1037/a0028085, 22506584

[ref35] Morgan M. J. Jr. Heo J. Osborn D. S. (2024). Career decision-making, career exploration behaviors, and self-regulated learning. Career Dev. Q. 72, 46–62. doi: 10.1002/cdq.12340

[ref36] Mulder J. D. (2023). Power analysis for the random intercept cross-lagged panel model using the powRICLPM R-package. Struct. Equ. Model. 30, 645–658. doi: 10.1080/10705511.2022.2122467

[ref37] Mulder J. D. Hamaker E. L. (2021). Three extensions of the random intercept cross-lagged panel model. Struct. Equ. Model. 28, 638–648. doi: 10.1080/10705511.2020.1784738

[ref38] Muthén B. Asparouhov T. (2024). Can cross-lagged panel modeling be relied on to establish cross-lagged effects? The case of contemporaneous and reciprocal effects. Psychol. Methods. doi: 10.1037/met0000661 38815066

[ref39] Newman D. A. (2014). Missing data: five practical guidelines. Organ. Res. Methods 17, 372–411. doi: 10.1177/1094428114548590

[ref40] Niu Y. Xu X. Lewis S. Xie L. Reed M. (2025). Examining the role of mentoring on perceived employability among university students in China. J. Career Assess. 33, 239–263. doi: 10.1177/10690727241256291

[ref41] Orth U. Meier L. L. Bühler J. L. Dapp L. C. Krauss S. Messerli D. . (2024). Effect size guidelines for cross-lagged effects. Psychol. Methods 29, 421–433. doi: 10.1037/met0000499 35737548

[ref42] Paganin G. Mazzetti G. Guglielmi D. Chiesa R. Mariani M. G. van der Heijden B. I. J. M. (2025). Exploring different types of university graduates: a latent profile analysis on career strategies and employability in the new labour market. Int. J. Adolesc. Youth 30:Article 2496451. doi: 10.1080/02673843.2025.2496451, 37339054

[ref43] Petruzziello G. Chiesa R. Guglielmi D. van der Heijden B. I. J. M. de Jong J. P. Mariani M. G. (2025). Self-perceived employability and psychological well-being among Italian students and graduates: a three-wave cross-lagged study. J. Career Dev. 52, 21–40. doi: 10.1177/08948453241296805

[ref44] Podsakoff P. M. Podsakoff N. P. Williams L. J. Huang C. Yang J. (2024). Common method bias: it’s bad, it’s complex, it’s widespread, and it’s not easy to fix. Annu. Rev. Organ. Psychol. Organ. Behav. 11, 17–61. doi: 10.1146/annurev-orgpsych-110721-040030

[ref45] Putnick D. L. Bornstein M. H. (2016). Measurement invariance conventions and reporting: the state of the art and future directions for psychological research. Dev. Rev. 41, 71–90. doi: 10.1016/j.dr.2016.06.004, 27942093 PMC5145197

[ref46] Qalati S. A. Badwy H. E. El-Bardan M. F. (2025). Career proactivity unleashed: charting the path to sustainable success through the art of self-regulated learning. J. Employ. Couns. 62, 116–126. doi: 10.1002/joec.12244

[ref47] Qalati S. A. Badwy H. E. Lathabhavan R. El-Bardan M. F. (2026). Human resources practices and career sustainability: analysis of multiple mediation mechanisms in Egyptian hotels. J. Hum. Resour. Hosp. Tour. 25, 219–246. doi: 10.1080/15332845.2025.2588518

[ref48] Rhemtulla M. Brosseau-Liard P. É. Savalei V. (2012). When can categorical variables be treated as continuous? A comparison of robust continuous and categorical SEM estimation methods under suboptimal conditions. Psychol. Methods 17, 354–373. doi: 10.1037/a0029315, 22799625

[ref49] Rodrigues R. van Harten J. De Cuyper N. Grosemans I. Butler C. (2024). On your marks, get set, go! Jumping the hurdles of employability development at an early career stage. J. Vocat. Behav. 151:103999. doi: 10.1016/j.jvb.2024.103999, 42574925

[ref50] Rohrer J. M. Murayama K. (2023). These are not the effects you are looking for: causality and the within-/between-persons distinction in longitudinal data analysis. Adv. Methods Pract. Psychol. Sci. 6:25152459221140842. doi: 10.1177/25152459221140842

[ref51] Stumpf S. A. Colarelli S. M. Hartman K. (1983). Development of the career exploration survey (CES). J. Vocat. Behav. 22, 191–226. doi: 10.1016/0001-8791(83)90028-3

[ref52] Tuononen T. Räisänen M. Hyytinen H. (2024). Students’ work experience in relation to their career engagement and metacognitive awareness. High. Educ. Res. Dev. 43, 1399–1415. doi: 10.1080/07294360.2024.2332251

[ref53] Usami S. (2021). On the differences between the general cross-lagged panel model and the random-intercept cross-lagged panel model: interpretation of cross-lagged parameters and model choice. Struct. Equ. Model. 28, 331–344. doi: 10.1080/10705511.2020.1821690

[ref54] Vanhercke D. De Cuyper N. Peeters E. De Witte H. (2014). Defining perceived employability: a psychological approach. Pers. Rev. 43, 592–605. doi: 10.1108/PR-07-2012-0110

[ref55] Vrieze S. I. (2012). Model selection and psychological theory: a discussion of the differences between the Akaike information criterion (AIC) and the Bayesian information criterion (BIC). Psychol. Methods 17, 228–243. doi: 10.1037/a0027127, 22309957 PMC3366160

[ref56] Whiston S. C. Li Y. Goodrich Mitts N. Wright L. (2017). Effectiveness of career choice interventions: a meta-analytic replication and extension. J. Vocat. Behav. 100, 175–184. doi: 10.1016/j.jvb.2017.03.010

[ref57] Xu H. Hou Z.-J. Tracey T. J. G. (2014). Relation of environmental and self-career exploration with career decision-making difficulties in Chinese students. J. Career Assess. 22, 654–665. doi: 10.1177/1069072713515628

