# Supplementary material for: Employment outlook before search? A four-wave RI-CLPM study of environmental career exploration and perceived employment outlook among undergraduates in Liaoning Province, China
Source: Front Psychol. 2026 Sep 4;17:1921600. doi: 10.3389/fpsyg.2026.1921600 (PMC13587606; doi:10.3389/fpsyg.2026.1921600)
Supplement: Supplementary file 1 [file Table_1.docx]

**Supplementary Materials**

**Table S1**

*Alternative measurement and missing-data specifications*

| **Model** | **Fit** | **Direction** | **T1→T2 β (p)** | **T2→T3 β (p)** | **T3→T4 β (p)** |
| --- | --- | --- | --- | --- | --- |
| Primary observed-score free-lag RI-CLPM with auxiliaries | χ²(9) = 48.333; CFI = .986; TLI = .956; RMSEA = .044; SRMR = .030 | EPO→ECE | .065 (.030) | .022 (.482) | .094 (.001) |
|  |  | ECE→EPO | −.003 (.914) | −.009 (.765) | −.069 (.024) |
| Observed-score free-lag RI-CLPM without auxiliaries | χ²(9) = 48.092; CFI = .986; TLI = .957; RMSEA = .044; SRMR = .030 | EPO→ECE | .065 (.031) | .023 (.479) | .094 (.001) |
|  |  | ECE→EPO | −.003 (.923) | −.009 (.765) | −.069 (.023) |
| Multiple-indicator free-lag RI-CLPM | χ²(566) = 805.830; CFI = .992; TLI = .991; RMSEA = .014; SRMR = .028 | EPO→ECE | .083 (.049) | .045 (.310) | .104 (.008) |
|  |  | ECE→EPO | .008 (.830) | −.027 (.529) | −.122 (.002) |
| Two-item EPO free-lag RI-CLPM with auxiliaries | χ²(9) = 40.535; CFI = .988; TLI = .964; RMSEA = .039; SRMR = .027 | EPO→ECE | .070 (.016) | .025 (.432) | .072 (.015) |
|  |  | ECE→EPO | .005 (.858) | −.011 (.734) | −.072 (.016) |

*Note.* ECE = environmental career exploration; EPO = perceived employment outlook; RI-CLPM = random-intercept cross-lagged panel model. All models used N = 2,267. The multiple-indicator model retained all three EPO items and allowed EPO3 intercepts to vary across waves. For the EPO1–EPO2 score, Spearman–Brown reliability was .755, .760, .765, and .733 at T1–T4. AIC and BIC were not compared across models with and without auxiliary variables because their likelihoods incorporated different observed-variable sets.

**Table S2**

*Stratified cross-group comparisons by university tier and academic major*

| **Grouping** | **Direction** | **Interval** | **Group A β (p)** | **Group B β (p)** | **p for difference** | **Holm-adjusted p** |
| --- | --- | --- | --- | --- | --- | --- |
| University tier | EPO→ECE | T1→T2 | .121 (.088) | .054 (.105) | .389 | .389 |
|  | EPO→ECE | T2→T3 | .111 (.108) | .003 (.934) | .167 | .334 |
|  | EPO→ECE | T3→T4 | .230 (.001) | .070 (.029) | .066 | .198 |
|  | ECE→EPO | T1→T2 | −.028 (.707) | .002 (.961) | .712 | .712 |
|  | ECE→EPO | T2→T3 | .098 (.180) | −.033 (.336) | .104 | .208 |
|  | ECE→EPO | T3→T4 | .072 (.317) | −.098 (.004) | .035 | .104 |
| Academic major | EPO→ECE | T1→T2 | .038 (.422) | .097 (.104) | .390 | .401 |
|  | EPO→ECE | T2→T3 | .096 (.055) | −.006 (.918) | .200 | .401 |
|  | EPO→ECE | T3→T4 | .121 (.005) | .015 (.814) | .127 | .380 |
|  | ECE→EPO | T1→T2 | −.010 (.820) | −.022 (.705) | .892 | 1.000 |
|  | ECE→EPO | T2→T3 | −.026 (.594) | .031 (.619) | .473 | 1.000 |
|  | ECE→EPO | T3→T4 | −.012 (.812) | −.063 (.285) | .535 | 1.000 |

*Note.* For university tier, Group A = Double First-Class (n = 342) and Group B = non-Double First-Class (n = 1,925). For academic major, Group A = STEM (n = 964) and Group B = humanities and social sciences (n = 558). Business, economics and management and arts and design students remained in the full-sample model but were excluded from the major comparison. Difference tests were based on unstandardized coefficients; standardized coefficients are shown. No interval-specific difference survived Holm correction.
